# A scoping review of how exposure to urban violence impacts youth access to sexual, reproductive and trauma health care in LMICs

**DOI:** 10.1080/17441692.2022.2103581

**Published:** 2022-08-07

**Authors:** Gill Green, Alison Swartz, Doreen Tembo, Diane Cooper, Asha George, Richard Matzopoulos, Andrea Fachel Leal, Cristiane Cabral, Regina Barbosa, Daniela Knauth

**Affiliations:** a School of Health and Social Care, University of Essex, Colchester, UK; b School of Public Health, University of Cape Town, Cape Town, South Africa; c Department of Psychosocial and Psychoanalytic Studies, University of Essex, Colchester, UK; d School for Healthcare Enterprise and Innovation, Faculty of Medicine, University of Southampton, Southampton, UK; e School of Public Health, University of the Western Cape, Cape Town, South Africa; f Division of Public Health Medicine, University of Cape Town, Cape Town, South Africa; g Burden of Disease Research Unit, South African Medical Research Council, Cape Town, South Africa; h Department of Sociology, Universidade Federal do Rio Grande do Sul, Porto Alegre, Brazil; i Department of Health, Life Cycles and Society, Universidade de São Paulo, São Paulo, Brazil; j Population Studies Centre 'Elza Berquó', Universidade Estadual de Campinas, São Paulo, Brazil; k Department of Social Medicine, Universidade Federal do Rio Grande do Sul, Porto Alegre, Brazil

**Keywords:** Urban violence, youth, health care access, LMIC

## Abstract

Violence in the community can impact access to health care. This scoping review examines the impact of urban violence upon youth (aged 15–24) access to sexual and reproductive health and trauma care in Low and Middle Income Countries (LMICs). We searched key electronic health and other databases for primary peer-reviewed studies from 2010 through June 2020. Thirty five of 6712 studies extracted met criteria for inclusion. They were diverse in terms of study objective and design but clear themes emerged. First, youth experience the environment and interpersonal relationships to be violent which impacts their access to health care. Second, sexual assault care is often inadequate, and stigma and abuse are sometimes reported in treatment settings. Third is the low rate of health seeking among youth living in a violent environment. Fourth is the paucity of literature focusing on interventions to address these issues. The scoping review suggests urban violence is a structural and systemic issue that, particularly in low-income areas in LMICs, contributes to framing the conditions for accessing health care. There is a gap in evidence about interventions that will support youth to access good quality health care in complex scenarios where violence is endemic.

## Introduction

Access to quality health services is a human right that is limited for many young people living in urban, low-income and violence-endemic neighbourhoods in Low and Middle Income Countries (LMICs). Our definition of urban violence is based upon a modified World Health Organisation (WHO) definition of violence which refers to ‘the intentional use of physical force or power, threatened or actual … that either results in or has a high likelihood of resulting in injury, death, psychological harm, maldevelopment, or deprivation’ (WHO, 2002, p. 4). We also draw upon representations of urban environments which highlight discourses of the city as a dangerous place because dangerous people live there (Body-Gendrot, 1995). Urban violence defined in this way affects many countries globally, particularly in low-income settings, with LMICs most severely affected (Matzopoulos et al., 2008).

Youth violence as defined by the WHO is a global public health problem (WHO, 2015) and in many low-income neighbourhoods violence is a structural part of everyday life, and young people, are disproportionally affected (de Ribera et al., 2019). Worldwide, homicides are the fourth leading cause of death for youth aged 10–29 (84% of whom are males) and one in eight young people (mainly girls) report sexual abuse (WHO, 2020).

The adverse impacts of violence not only include mortality, direct injury/physical trauma but also a deterioration of mental health and well-being that may in turn impose a direct (and measurable) burden on the health system, while at the same time driving rates of violence even higher within afflicted communities (Matzopoulos et al., 2008). Moreover, perceived fear and exposure to community violence is associated with a decreased likelihood of seeking care (Mmari et al., 2016). Urban violence can impede access to care and also negatively impact the operation of an effective health system (Bowers, 2008; Cooper et al., 2019; Michelman & Patak, 2008). Violence is thus a barrier to universal health coverage by inhibiting youth utilisation and access to acceptable health care.

This scoping review aimed to improve understanding of the barriers (and ways to overcome such barriers) created by urban violence on youth health care access. There is no available review of youth access to care in the context of violence in LMICs. This review thus complements the existing literature about the association between violence and health, the great majority of which is based on studies conducted in high-income countries rather than in LMICs. This includes: health systems response to violence against women, particularly to support women subjected to intimate and sexual violence (García-Moreno et al., 2015); the impact of stigma on care-seeking (Kinsler et al., 2007); literature on aspects of youth violence (e.g. Involvement in gangs, violence and place, negative health consequences of violence), much of which focuses on violence prevention (Nation et al., 2021).

We sought to review the available evidence in order to identify key characteristics or factors related to urban violence and youth access to sexual, reproductive and trauma care. We sought to answer the question: What is known about how urban violence impacts access to and delivery of health care for sexual and reproductive health and trauma services for youth in LMICs?

Our focus is on violence in public spaces rather than in domestic settings, which we categorise into three broad and intersecting forms. Our first category is interpersonal violence in both physical and psychological domains – either directly as victims or perpetrators themselves or as members of communities where such violence is highly prevalent. Gender-based violence including sexual assault and rape are implicitly included as their effects span both the physical and psychological domains. Our second category is community violence as defined by Wright and colleagues (2016). This refers to violence occurring outside the home but does not include acts of war and terrorism. Both victimisation by community violence (i.e. being the recipient of an intentional act intended to cause harm) and witnessing community violence (i.e. seeing an event that involves loss or injury of some kind, including death) are included. Our final category is institutional/systemic violence within the health system. Institutional violence includes expressions of racism and disrespect to cultures and ethnicities (Fernandes et al., 2018) as well as stigma and discrimination (see Parker, 2012), which can be experienced and perpetuated at individual and community levels.

This review synthesises the evidence base we identified and provides valuable insights for the design of future interventions to limit the impact of urban violence on access and delivery of health care for young people.

## Method

A scoping review was selected rather than a systematic review as the purpose was to review a broad body of literature to identify knowledge gaps and identify key characteristics related to the concept (Munn et al., 2018). Scoping reviews are well suited to address explanatory research questions, identify key concepts and enable the use of broad inclusion criteria to identify a range of available evidence (Arksey & O’Malley, 2005).

We focused on sexual and reproductive health (SRH) and trauma services as these are most frequently sought by young people. In addition, sexual assault (primarily affecting females) and trauma (primarily affecting males) are risks for youth exposed to violence. SRH includes a range of treatment and care services (contraception, abortion, maternal health, sexually transmitted infections, HIV testing and treatment, reproductive tract infections, sexual violence, menstrual problems). Trauma includes services dealing with wounds and other physical injuries as well as psychological trauma.

### Search strategy

A combination of keywords to identify articles including youth, violence and health care were developed by the authors (see Supplementary Material: Appendix 1). The following electronic databases were searched: PUBMED; SCI Web of Science; SCOPUS; the Medical Literature Analysis and Retrieval System Online (MEDLINE); PsycINFO; Cochrane Central Register of Controlled Trials (CENTRAL); Database of Abstracts of Reviews of Effectiveness (DARE) NLM Gateway;, the Cumulative Index to Nursing and Allied Health Literature (CINAHL); LILACS; the Educational Resources Information Center (ERIC). The search was performed in early July 2020.

### Eligibility criteria

This review was conducted as part of a grant for research about health care systems in LMICs as defined by the World Bank classification. Our decision to limit the review to LMICS was not only made in relation to the funding body but also because LMICS face different challenges to high-income countries in relation to health systems, services and general social context. The focus on LMICS would thus allow for more meaningful analysis of challenges and responses. The impact of urban violence is likely to vary according to resources available to health care systems and there is a relative dearth of LMIC literature despite the disproportionate burden of interpersonal violence on health and development in LMICs. We were also keen to explore contemporary experiences and were mindful that urban violence contexts change over time as the country contexts (health system, characterisation of violence, programmes and interventions) around which issues of violence operate are likely to change significantly over a period of ten years, We, therefore, restricted our review to papers published between January 2010 and June 2020 and included those that met the following criteria: based on primary research; focused on LMICs; focused on youth aged 15–24 (at least two-thirds of the sample or the article presented age-specific results); were conducted in an urban setting or had a predominantly urban-dwelling sample (at least two-thirds of the sample); focused on SRH treatment, prevention (but not health promotion) or care and trauma services; included violence outside the domestic setting; were written in English, Portuguese or Spanish (languages in which the research team were proficient).

We excluded papers that: focused exclusively on intimate partner and/or domestic violence; state violence; poverty; inequality; health promotion; war; cyber victimisation, self-harm (including suicide/suicidal ideation).

### Article selection

The first two authors reviewed titles and abstracts of all the references and identified candidate articles for full-text review. The first author reviewed the full-text articles to determine inclusion in the review based on parameters that were developed and refined in discussions between the first three authors.

### Data extraction

The first author extracted the following data for all included studies: aims, location, type of violence, type of service sought, details of any intervention, study population, method, key relevant findings.

### Data analysis

The characteristics of the studies were first summarised (see Table 1) and presented descriptively to illustrate the type and scope of the included literature. As the studies we identified were extremely heterogeneous both in terms of focus and method used, we used a thematic synthesis approach to summarise the substantive findings. We were broadly guided by a socio-ecological model (Brofenbrenner, 1979) where individual, interpersonal, organisational, community and systems level factors interact to impact health care access. Initially the first two authors extracted study findings that reported an association between urban violence and health care access. We then used an inductive approach to identify ten preliminary themes. We mapped these against a conceptual framework of access to health care (Levesque et al., 2013) that explores: health care needs; perception of need and desire for care; health care seeking; health care reaching; health care utilisation; health care consequences. In attempting to map our findings against this framework, we realised that several themes needed to be further synthesised. Hence some were expanded and others collapsed into broader thematic categories. This process was undertaken initially by the first 3 authors and later involved the broader interdisciplinary research team, that includes perspectives from public health, health systems, sociology and anthropology. The final agreed themes are presented in the results section.

**Table 1.** Details of studies included in the scoping review.

Number, first author and year	Primary objective	Study location	Type of violence	Service being accessed	Study population	Method
1. Abrahams et al. (2010)	To test a psychosocial intervention to increase post-exposure prophylaxis (PEP) adherence	3 urban sexual assault centres in the Western Cape and one in the Eastern Cape, South Africa	Interpersonal: Rape	Hospital for post-rape care	279 female rape survivors (over 75% under 23 years old) prescribed PEP	Quant: RCT
2. Akinlusi et al. (2014)	To assess the characteristics of sexual assault survivors and treatment offered	Lagos, Nigeria	Interpersonal: Sexual assault	Hospital for post- assault care	287 females aged 2–50 (mean age 12.9) presenting following sexual assault	Quant: medical records
3. Badejoko et al. (2014)	To determine the burden, presentation and management of sexual assault against women	Ile-Ife, Nigeria	Interpersonal: Sexual assault	Hospital for post assault care	71 females aged 7–50 (69% aged 11–25 and some younger)	Quant: medical records
4. Baron et al. (2020)	To evaluate peer-based clubs offered to young women to support Pre-exposure prophylaxis (PrEP) adherence	A high-density inner-city neighbourhood in Johannesburg, South Africa	Interpersonal: Non-specific gender- based violence	Clubs to encourage HIV prevention	13 women aged 18–24 years (median age 21 years) who attended EMPOWER clubs	Qual: interviews at baseline and follow-up
5. Bohren et al. (2019)	To develop tools to measure and report mistreatment during childbirth in four LMICs	12 health facilities in urban areas in Nigeria, Ghana, Guinea and Myanmar	Institutional: Physical or verbal abuse or stigma by health care workers (HCWs)	Hospital for labour	2016 labour observations and 2672 surveys of women over 15 years admitted for childbirth	Quant: observation and survey
6. Burke et al. (2017)	To explore the barriers and enablers for young people with disabilities to access Sexual and Reproductive Health (SRH) services	Dakar (urban), and Thies and Kaolack (areas with urban and rural areas) in Senegal	Interpersonal and Institutional: Sexual violence and provider attitudes	None	144 young people aged 18–24 years living with a physical or sensory impairment	Qual: focus groups and interviews
7. Daru et al. (2011)	To document the clinical findings of women seeking care post-rape	An urban teaching hospital in Jos, Nigeria	Interpersonal: Rape	Hospital post rape	105 women aged 1–24 (mean age of 12 years) presenting following rape	Quant: medical records
8. Deschamps et al. (2019)	To describe the characteristics of sexual assault and its psychological consequences among female victims	Port au Prince, Haiti	Interpersonal: Sexual assault	Hospital post sexual assault	4092 females (median age 19) who presented at the clinic following sexual assault	Quant: medical records
9. Figueiredo et al. (2012)	To examine the use of free distribution of emergency contraception to adolescents	Municipality of Sao Paolo Brazil which is mainly urban	Interpersonal: Sexual violence	Emergency contraception	101/119 municipalities in the state of São Paulo	Quant: survey
10. Garcia (2015)	To describe ‘treatment’ centres run by marginalised populations and what this reveals about recovery	Working class neighbourhood in Mexico City	Institutional: Violence in the treatment setting	Informal residential treatment	18 residents with long term drug and mental health problems	Qual: Ethnography
11. Gatuguta et al. (2018)	To compare the characteristics of survivors who present for healthcare to identify barriers to treatment	Naivasha and Thika towns, Kenya	Interpersonal: Sexual violence	Hospital post sexual violence	543 survivors (69% were under 18) of sexual assault who presented to hospital	Mixed: medical records and interviews with HCWs
12. Harrison et al. (2017)	To describe the differences between clients presenting after rape and clients who have consented to sex as a minor, and how these differences affect their care requirements	A low-income suburb of Harare, Zimbabwe	Interpersonal: Rape	NGO clinic for post rape care	3617 clients (93% female and 70% <16 years) presenting to the clinic	Quant: client records
13. Leeper et al. (2017)	To report characteristics and opportunities for intervention of assault-injured youth	Emergency centres in a low-income neighbourhood in Cape Town South Africa	Interpersonal: Assault	Emergency treatment centres	513 patients aged 14–24 years old (324 assault-injured patients (80% male) and 189 controls)	Quant: survey
14. Lince-Deroche et al. (2015)	To assess young women’s SRH knowledge and experiences and to determine how they get SRH services	A township in Soweto in Johannesburg municipality, South Africa	Interpersonal and Institutional: Gender-based violence and provider attitudes	SRH services	90 women aged 19–23 recruited from primary care clinics and shopping malls	Mixed: survey and interviews
15. Luffy et al. (2019)	To understand young women’s personal experiences of unintended pregnancy	A city in North Central Nicaragua	Interpersonal and Institutional: Rape, criminalisation of abortion and provider attitudes	Unsafe Abortion	1 woman aged 19 pregnant following rape	Qual: case study
16. Macleod and Feltham-King (2020)	To outline a reparative justice/care approach to adolescent reproductive health	A township in Eastern Cape, South Africa	Interpersonal and Institutional: Rape, unwanted pregnancy and provider attitudes	Ante-natal care	5 young pregnant women living in a township	Qual: ethnography and interviews
17. Maclin et al. (2020)	To elucidate impact that gender-based violence and fear have on the very poor in rapidly growing cities and their coping strategies	Low-income urban areas in Bangladesh (Dhaka), Haiti (Port-au-Prince), Ethiopia (Addis Ababa)	Interpersonal and Community: Violence and fear of violence particularly for women	General health care	105 urban poor + 11 key informant (professional) interviews	Qual: Focus groups and interviews
18. Mmari et al. (2016)	To examine the influence of the social context on adolescent health care seeking	Nigeria (Ibadan) South Africa (Johannesburg), India (New Delhi), China (Shanghai), U.S.A. (Baltimore)	Community: Witnessing community violence	General health care including SRH	2393 disadvantaged urban adolescents aged 15–19 years	Mixed: survey and range of qual methods
19. Moss et al. (2019)	To present contextual perspectives of girls living on the streets and the implications for the delivery of services	Abidjan, Cote d’Ivoire	Interpersonal: physical and sexual violence	Pharmacy for SRH medication	11 women aged 14–18 living on street and engaged in sex work	Qual: interviews
20. Muriuki et al. (2017)	To evaluate the characteristics of survivors of sexual violence and review the uptake, adherence, and outcomes of those initiated on PEP	Nairobi, Kenya	Interpersonal: Sexual assault	Hospital post rape care	385 assaulted persons in area of high HIV prevalence; 86% were female; the median age was 21.	Quant: medical records
21. Myers et al. (2016)	To explore perceptions of factors that influence poor Alcohol or Drug (AOD) using young women’s use of health services	Two peri-urban townships in` Cape Town, South Africa	Interpersonal Community and Institutional: Witnessing and experiencing gang violence in community and exclusion from care	General health services (mainly primary)	23 women (aged 16–21) who use AODs in 2 townships Also, 14 in-depth interviews with service planners and providers	Qual: focus groups and interviews
22. Navarro et al. (2012)	To establish the prevalence of exposure to physical and sexual violence and association with treatment-seeking behaviour among street-based subpopulation	Tegucigalpa, Honduras	Interpersonal: Physical and sexual violence	A medical facility	Street-based populations: 59 male and 22 females aged 10-18; 153 adults and 49 sex workers.	Quant: survey
23. Pantelic et al. (2020)	To examine discrimination and retention in care among adolescents living with HIV	Resource-limited district in the Eastern Cape South Africa (73% of sample identified as urban)	Institutional: Discrimination from HCWs	HIV adherence	1059 adolescents living with HIV and 979 in follow up interview	Quant: survey at baseline and 18 months later
24. Place et al. (2019)	To examine pathways to care of women survivors of sexual assault	6 states (5 predominantly urban) in Guatemala	Interpersonal and Institutional: Sexual assault including rape and care pathway	Post-rape care	23 women who sought care following abuse (14/18 who were asked their age were <24 years)	Qual: Interviews
25. Renzaho (2017)	To examine factors associated with SRH including gender-based violence	Urban slums in Kampala Uganda	Interpersonal: Sexual coercion including rape	None	663 participants aged between 13 and 24 years living in urban slums	Quant: survey
26.Ritchwood et al. (2019)	To identify aspects of the clinic environment that either improve or inhibit adolescents living with HIV’s engagement in care	Local HIV clinics in Cape Town, South Africa	Institutional: Negative attitudes of HCWs	HIV treatment	Adolescents living with HIV 13–19 years old (*n* = 20), their caregivers (*n* = 19), and local stakeholders (*n* = 20)	Qual: interviews
27. Robert et al. (2020)	To identify enablers and barriers in accessing HIV and SRH services among adolescent key population	Mainly urban counties (Nairobi, Mombasa) and Kisumu (mixed rural and urban), Kenya	Institutional: Violence and discrimination from HCWs	HIV and SRH services	108 vulnerable adolescents aged 10–19: boys reporting same sex relations; girls engaging in sex work; and adolescents injecting drugs	Qual: focus groups and interviews
28. Roberts et al. (2020)	To estimate the burden of vulnerabilities related to gender equality and extent of contact young sex workers have with programmes to support them.	Sex worker venues in Mombasa, Kenya	Interpersonal: Physical and sexual	None	408 young women aged 14–24 who sell sex	Quant: survey
29. Sarnquist et al. (2014)	To evaluate the efficacy of an empowerment and self-defense intervention for adolescent girls to decrease the incidence of sexual assault and harassment	Informal settlements near Nairobi Kenya	Interpersonal: Rape	None	Adolescent girls aged 13–20 from poor areas. 1798 in empowerment group and 428 in life skills group	Quant: survey at baseline and 10.5 months later
30. Sawyer-Kurian et al. (2011)	To understand the cultural contexts of the risks for adolescent females who have dropped out of school	Townships in Cape Town, South Africa	Interpersonal: Rape and general violence	None	37 Black and Coloured female school drop outs aged 13–17	Qual: Focus groups
31. Scorgie et al. (2017)	To understand the forms of interpersonal violence experienced by adolescents: to explore how violence is experienced differently by boys and girls; how they conceptualise ‘dangerous’ and ‘safe’ spaces; to identify gaps in available services	A low-income neighbourhood of Johannesburg, South Africa	Community and Interpersonal: Witnessing or experiencing community violence	NGOs working with youth	59 15–19-year-olds exposed to violence (as victim or witness) + 17 key informant interviews with youth workers	Qual: interviews and community mapping
32. Selenga and Jooste (2015)	To describe the experiences of youth victims of violence attending a community health centre	A low-income area of Cape Town South Africa	Interpersonal and Community: Victims of physical violence in the community	Community health centre	8 men aged 18–27 who were victims of physical attack in the community	Qual: interview
33. Souza da Silva et al. (2019)	To understand the feelings experienced by health professionals caring for young victims of violence	A city in Rio Grande do Sul, Brazil	Interpersonal: Victim of violence (not defined)	Mental health service	10 health professionals in a child mental health service	Qual interviews
34. Taquette et al. (2017)	To analyse the structure of healthcare that provide SRH services to the adolescent population	Outpatient clinics in Rio de Janeiro, Brazil	Interpersonal: Sexual violence	SRH care	147 coordinators of SRH outpatient clinics treating young people aged 13–19	Quant: survey
35. van Wyk and Davids (2019)	To report on adherence challenges faced by adolescents receiving ART	Low-income township in Cape Town, South Africa	Institutional: Hostile and discriminatory caregivers	HIV treatment adherence	15 adolescents with HIV living in a low socio-economic urban setting	Qual: focus groups and interviews

## Results

### Study selection

**Figure 1.**
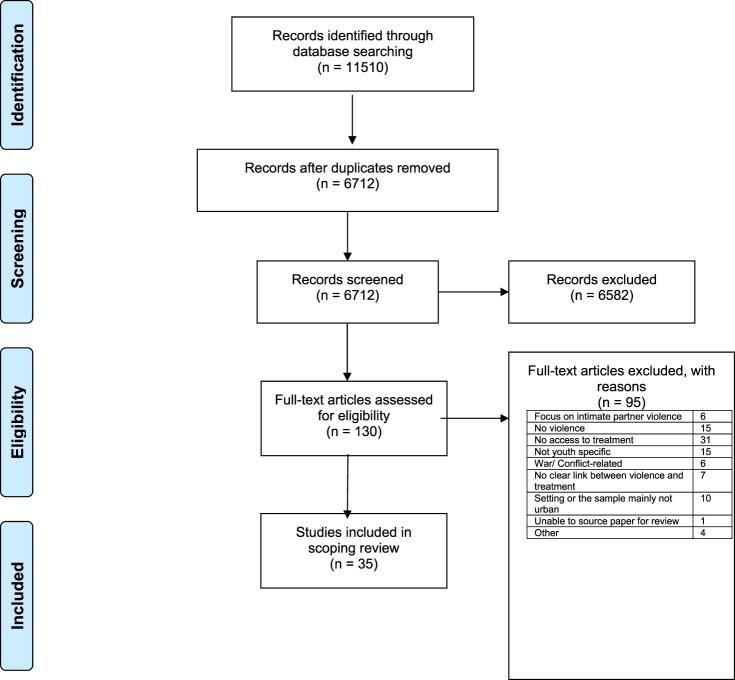
PRISMA flowchart of included studies.

The search identified 11,510 citations, and, following the removal of duplicates, a total of 6712 titles and abstracts were screened for relevance. A total of 6582 articles were excluded at this stage and 130 full-text articles were appraised for eligibility. Of these 95 were excluded, based on assessment using the criteria described above, with 35 articles included in the scoping review (Figure 1).

### Characteristics and study designs

The studies that met the inclusion criteria following the screening process were heterogeneous in relation to: context and population, study objective, violence reported, type of care, and methodological approach.

Over two-thirds (24) of the studies were based in Africa (including 12 from South Africa) and 5 from Central America, 3 from Brazil and 3 based on multi-country studies. There was heterogeneity in the primary objectives of the studies but all included a direct link between experience of interpersonal, community or institutional violence and seeking or receiving health care (mainly SRH or trauma care). In terms of our categorisation of violence, 28 were about interpersonal violence (Table 1 nos. 1, 2, 3, 4, 6, 7, 8, 9, 11, 12, 13, 14, 15, 16, 17, 19, 20, 21, 22, 24, 25, 28, 29, 30, 31, 32, 33, 34), 20 of which included sexual assault or rape. Five were about community violence (Table 1 nos. 17, 18, 21, 31, 32). Twelve were about institutional/systemic violence (Table 1 nos. 5, 6, 10, 14, 15, 16, 21, 23, 24, 26, 27, 35). Nine studies included more than one category including one study that reported findings related to all three categories (see Table 1 nos. 6, 14, 15, 16, 17, 21, 24, 31, 32). The care categories included: 11 receiving post rape/sexual assault or other emergency trauma treatment (Table 1 nos. 1, 2, 3, 7, 8, 11, 12, 13, 20, 24, 33); 5 HIV treatment or prevention (Table 1 nos. 4, 23, 26, 27, 35); 8 seeking SRH care (Table 1 nos. 5, 9, 14, 15, 16, 18, 19, 34); 6 seeking generic health care (Table 1 nos. 10, 17, 21, 22, 31, 32); 5 receiving no care with the articles focussing on barriers to care seeking (Table 1 nos. 6, 25, 28, 29, 30).

The study populations included young people in the general population mainly those living in low-income neighbourhoods, as well as some specific sub-populations: people living with HIV, sex workers, substance misusers, those with disabilities.

The body of literature is characterised by diverse disciplinary approaches from health services research to psychology and draws on a variety of methodological approaches and study designs. In total, there were 16 quantitative studies (including 1 randomised control trial, 9 based on (mostly cross-sectional) surveys, 6 based on analysis of medical records); 16 qualitative studies (13 using focus groups and/or interviews, 2 ethnographic methods and 1 case study) and 3 using mixed methods.

### Themes identified

In relation to the research question what is known about how urban violence impacts access to and delivery of health care for youth, themes were identified. These are displayed in Table 2 which also links each theme with the associated papers.

**Table 2.** Themes and papers.

Theme	Paper which addressed this (first author named only)
(1) Contexts of violence experienced by youth and impact on access	Lince-Deroche et al. (2015), Luffy et al. (2019), Maclin et al. (2020), Macleod and Feltham-King (2020), Myers et al. (2016), Navarro et al. (2012), Renzaho et al. (2017), Roberts et al. (2020), Sawyer-Kurian et al. (2011), Scorgie et al. (2017)
(2) Services that are available are inadequate *Sub theme: Stigma and abuse in treatment settings Sub theme: Sexual-assault care provided generally does not meet need*	Mmari et al. (2016)
	Bohren et al. (2019), Burke et al. (2017), Garcia (2015), Lince-Deroche et al. (2015), Macleod and Feltham-King (2020), Pantelic et al. (2020), Ritchwood et al. (2019), Robert et al. (2020), van Wyk and Davids (2019)
	Akinlusi et al. (2014), Daru et al. (2011), Figueiredo et al. (2012), Gatuguta et al. (2018), Muriuki et al. (2017), Place et al. (2019), Selenga and Jooste (2015), Souza da Silva et al. (2019), Taquette et al. (2017)
(3) Low rates of health seeking *Sub theme: There is limited knowledge about SRH services*	Akinlusi et al. (2014), Badejoko et al. (2014), Daru et al. (2011), Deschamps et al. (2019), Harrison et al. (2017), Mmari et al. (2016), Muriuki et al. (2017), Navarro et al. (2012), Place et al. (2019), Sawyer-Kurian et al. (2011)
	Burke et al. (2017), Moss et al. (2019), Renzaho et al. (2017), Roberts et al. (2020)
(4) Insufficient attempts to address inadequate services/low uptake	Abrahams et al. (2010), Leeper et al. (2017), Sarnquist et al. (2014)

#### Theme 1: Contexts of violence experienced by youth shape access to services

Structural contexts, including social, political, economic, historical and geographical forces, influence the use of health services. For example, poverty interacts with gender power relations and undermines good reproductive outcomes (Macleod & Feltham-King, 2020). Young women who use alcohol or drugs often do not seek support services as they cannot see the potential for their lives to improve. They have also often experienced mistreatment such as exclusion and stigma from service providers (Myers et al., 2016). Social norms and (often restrictive and conservative) national laws impact on adolescents’ health decisions resulting from violence and unintended pregnancy whereby the criminalisation of abortion can result in unsafe abortion (Luffy et al., 2019).

All the papers included in the scoping review allude to a context in which violence is commonly experienced by young people, yet there is evidence that many do not access care. In a study of adolescents in a low-income area of Johannesburg, 67% of males and 48% of females reported being a victim of violence in the past 12 months. Yet they were generally either unaware of how or where to access support services or doubted their ability to meet their needs (Scorgie et al., 2017). In another South African study, 28% of the young women surveyed reported that abuse, sexual harassment and rape were one of their most common life concerns, but 9% indicated that they did not know where to go for help (Lince-Deroche et al., 2015). Sexual abuse was reported among young people living in an urban slum in Kampala with 34% affirming that it was alright for a boy to force a girl to have sex if he had feelings for her and 73% affirming that it was common for strangers and relatives to force young females to have sexual intercourse with them without consent (Renzaho et al., 2017). However, less than half (48%) had visited a health facility to obtain information about contraception and sexually transmitted infections in the preceding 12 months; and the proportion was significantly lower for younger participants (37.3% vs. 52.8%, *p* < 0.01).

Within the broader socio-structural context, some groups are particularly vulnerable. In Honduras, among street-based adolescents aged 10–18, 59% self-reported exposure to physical violence in the last year and 44% sexual violence. Yet they were much less likely than adults to access care, with 50% seeking care following severe physical assault and only 14% seeking treatment following severe sexual assault (Navarro et al., 2012). Violence, rape, including by gangs (often drug use related) was a key theme reported in focus groups with teenagers in Cape Town who had dropped out of school (Sawyer-Kurian et al., 2011), with many women saying they would not disclose rape, including gang rape, due to fear of the perpetrator or other gang members. Among young women who sell sex in Mombasa 30% and 29% respectively had experienced physical and sexual violence, yet there was little awareness of programmes providing support (Roberts et al., 2020).

A study of urban poor living in three cities in three separate continents found that violence and/or fear of violence had significant impact on health and well-being, particularly for women. Participants in all three cities described a variety of health issues arising directly from violence, including physical and mental trauma (Maclin et al., 2020). They also reported that violence and coping strategies to avoid violence constrained mobility resulting in restricted access to health care. For example, community members in Port-au-Prince said it was unsafe travelling at night and they had to wait until morning to visit a clinic. Respondents in Dhaka said they sometimes avoided seeking health care as the men who worked at the service made them feel unsafe (Maclin et al., 2020).

#### Theme 2: Services that are available are inadequate

There is a need for young people to access safe spaces and efforts to connect adolescents to health care need to build trust (Mmari et al., 2016). However, a key theme in the literature was that services often failed to provide this.

*Stigma and abuse in treatment settings.* A study of an informal residential treatment centre in Mexico City run by marginalised populations shows how violence and care can co-exist (Garcia, 2015). Stigma and violence are also reported in formal treatment settings. A study covering four countries reports high rates of physical abuse, verbal abuse, and stigma during childbirth, particularly among younger age groups (Bohren et al., 2019). Young pregnant women in a South African township reported poor provider attitudes and numerous health system failures (Macleod & Feltham-King, 2020).

Stigma and discrimination can act as a barrier to care. Provider attitudes were cited as a main barrier to SRH care among people with disabilities (Burke et al., 2017). Young women recruited from a general population in Soweto, South Africa reported providers unsupportive attitudes and anticipated stigma to be a barrier to accessing SRH services (Lince-Deroche et al., 2015). For vulnerable young people living with HIV, enablers to access SRH and HIV services were privacy and confidentiality and limited stigma and discrimination, and barriers included negative attitudes from health providers (Ritchwood et al., 2019; Robert et al., 2020). Fear of unintended disclosure of HIV status, stigma and discrimination and treatment fatigue negatively influenced adherence in a low-income area in Cape Town (van Wyk & Davids, 2019) and can lead to non-retention in HIV care (Pantelic et al., 2020).

*Sexual-assault care provided generally does not meet need.* Provision of post rape care includes post-exposure prophylaxis to HIV (PEP) and/or HIV testing and counselling and sometimes emergency contraception (Akinlusi et al., 2014; Daru et al., 2011; Gatuguta et al., 2018; Muriuki et al., 2017). There is evidence that adolescents receive less care than older people (Figueiredo et al., 2012; Gatuguta et al., 2018) and, with some exceptions (Selenga & Jooste, 2015), generally do not receive care needed to address the full range of needs (Place et al., 2019).

The literature suggests that there is a range of reasons underlying the inadequate provision of post sexual assault care, particularly for young people. In Rio Grande do Sul in Brazil, professionals treating victims of violence feel powerless due to lack of resolution of cases and delays in referrals (Souza da Silva et al., 2019). Other studies report: limited availability of PEP or other equipment (Gatuguta et al., 2018); poor coordination and/or organisation of services including a lack of training among health care workers (Daru et al., 2011; Figueiredo et al., 2012; Gatuguta et al., 2018; Taquette et al., 2017). Stigma is also reported (Gatuguta et al., 2018) and prejudice towards adolescent sexual practices and abortion (Figueiredo et al., 2012; Place et al., 2019).

#### Theme 3: Low rates of health seeking

A study of adolescents living in low-income urban settlements in five cities across the world found that many do not disclose abuse and/or seek health care often linked to embarrassment, perceived stigma and a lack of trust in the services (Mmari et al., 2016). In Johannesburg, more than 30% of adolescents did not seek care even when they knew it was needed. Perceived fear and exposure to community violence was associated with a decreased likelihood of seeking care (Mmari et al., 2016). Not seeking support was also linked to gender inequity, whereby women may be stigmatised if they were raped, which may prevent them disclosing or seeking service support (Sawyer-Kurian et al., 2011).

Delayed presentation of young people to health services is also reported. Studies based on retrospective analysis of medical records suggest that delayed presentation post sexual assault is common (Akinlusi et al., 2014; Badejoko et al., 2014; Daru et al., 2011; Place et al., 2019). Young people are less likely to seek help and more likely to report later than older people (Daru et al., 2011; Deschamps et al., 2019; Navarro et al., 2012). Those more likely to present earlier are those experiencing physical violence during assault (Deschamps et al., 2019; Harrison et al., 2017) and those assaulted by an unknown person (Harrison et al., 2017). Perhaps linked to the inadequacy of much of the care provided, low rates of adherence with sexual assault care are reported (Muriuki et al., 2017), with, for example, only 13% of survivors returning for follow-up from a hospital in Nigeria (Badejoko et al., 2014).

*There is limited knowledge about SRH services.* A lack of knowledge of SRH services is reported among particular groups. Young people with disabilities reported very low knowledge about, and use of, SRH services with only 9 out of 50 interviewed ever having accessed SRH services (Burke et al., 2017). There was limited knowledge about SRH services among young females living on the street in Cote D’Ivoire and seeking medication from street vendors or a pharmacy was often used for unintended pregnancy, abortion and other SRH concerns (Moss et al., 2019). The majority (77%) of young people living in an urban slum in Uganda knew where and how to access contraception, but the proportion was significantly lower among 13–17 year old participants than those who were older (Renzaho et al., 2017). Among young women who sell sex, only 26% were aware of any programmes providing services to female sex workers (Roberts et al., 2020).

#### Theme 4: Insufficient attempts to address inadequate services/low uptake

Interventions that build young peoples’ social capital and resilience are essential for reducing violence-related trauma and long-term health and social consequences (Scorgie et al., 2017). There are opportunities to target interventions at high risk youth in health care settings as, for example, almost half (47%) of assault-injured youth in emergency centres in Cape Town reported a history of fighting requiring medical treatment in the previous six months (Leeper et al., 2017). However, this scoping review identified only three interventions in place to address these issues. Two aimed to enhance youth access to care through developing their empowerment and/or resilience. In a low-income neighbourhood in Johannesburg, a peer club using a structured empowerment approach improved use of Pre-Exposure Prophylaxis to HIV and increased self-efficacy and self-esteem (Baron et al., 2020). In Kenya, empowerment classes for adolescent girls led to a significantly (*P* = .001) decreased rate of sexual assaults at follow-up and an increased rate of disclosure from 56% to 75% (*P* = .006) (Sarnquist et al., 2014). In South Africa, a randomised control trial of provision of telephonic psychosocial found that it did not significantly improve PEP adherence among rape survivors (Abrahams et al., 2010).

## Discussion

This scoping review provides evidence and valuable insights about how exposure to urban violence impacts youth access to sexual, reproductive and trauma health care in LMICs – a topic about which there have not been other reviews.

Two-thirds of the 35 articles we identified were based in Africa including 12 from South Africa and there were 5 from Central America and 3 from Brazil reflecting the high levels of urban violence in these areas in the global South (Salahub et al., 2019). The majority of articles focused on SRH including sexual assault rather than physical trauma and there were more that focused on young women than men.

There was diversity in terms of primary aims, methods and samples but some clear themes nevertheless emerged. The scoping review has confirmed that young people living in low-income urban settings experience their environments and interpersonal relationships as violent and this has a negative impact of their access to health care. All the papers reviewed either presented evidence or implied that violence constituted a structural part of everyday life. They illustrated how violence in the community operates, e.g. through conflicts involving drug dealers, gangs or local police. These are portrayed as sporadic and normalised (Scorgie et al., 2017). This suggests that there is a need to focus on power relations and structural inequalities, rather than individual responsibility in terms of understanding and addressing youth access to health care. Poverty interacts with gender power relations in a way which undermines young women’s pursuit of sexual and reproductive justice (Macleod & Feltham-King, 2020). Gender inequality and stigma toward young women who use alcohol or drugs leads to their social exclusion from education and employment opportunities and health care (Myers et al., 2016). Urban violence and structural inequalities can limit how young people move about the community and restricts access to health care (Maclin et al., 2020). It is also linked to desensitisation to violence whereby violence is not always reported or recognised as such with high rates of non-disclosure of violence and non-presentation or late-presentation to health care (Navarro et al., 2012). Young people may fail to seek care when they need it often because of a lack of trust in providers or embarrassment or feeling stigmatised for seeking services (Mmari et al., 2016).

They may also be deterred by the quality of health care services, which in many cases were inadequate. Institutional violence within health services, often in the form of stigma, discrimination and/or hostile attitudes towards young people emerged as a key issue (Lince-Deroche et al., 2015). Youth report that they do not feel welcome and are discriminated against, feeling that health professionals have a moralistic reproach to what they do or say (Burke et al., 2017; Lince-Deroche et al., 2015). This may impact on youth access to services and on adherence to treatment (Ritchwood et al., 2019).

Vulnerability in terms of violence’s impact on sexual and mental health requires an intersectional understanding and integrated approach to providing health services (Moss et al., 2019). Despite this, and the barriers to young people accessing health care in areas with high levels of urban violence, there is a paucity of literature based on interventions that address the impact of violence on health care access in LMIC settings. The three studies we identified (Abrahams et al., 2010; Baron et al., 2020; Sarnquist et al., 2014) all focused on modifying the behaviour of individuals. The ubiquity of violence in some low-income urban environments, which impacts on both young people and those involved in the delivery of care, would suggest that a whole-system approach would be most effective.

There are several examples of intervention studies to draw on reported in systematic reviews or in non-LMIC settings, which due to our criteria for papers based on primary data, were not included in our review. For example, a systematic review of studies of SRH interventions for young people in LMIC and humanitarian settings suggests preliminary support for the effectiveness of several evidence-based SRH interventions targeting young people in humanitarian and low resource settings. However, there were a number of methodological weaknesses identified in this body of literature, e.g. short follow-up periods and few studies that focused on the feasibility, implementation and sustainability of interventions on a broad scale (Desrosiers et al., 2020). A systematic review of intervention programmes conducted across the globe (not just LMIC settings) targeting gender inequality among young people found that they generally focus on improving individual agency, rather improving broader systems (Levy et al., 2020). There is, however, recognition that multisectoral perspectives are required to address youth violence (Bolton et al., 2017).

### Limitations

A major limitation of this scoping review was the breadth of the literature searched, due to the exploratory nature of our project. We identified a vast range of different types of studies focusing on varying aspects of urban violence and youth access to health care. This made summation a challenge as the studies were not comparing like with like and their aims and outcomes were very varied. In addition, our requirement that papers had to include links between urban violence and youth health care access meant that there were many papers of interest about either violence or health care access alone that we could not include. Most notably were the many intervention studies that aimed to tackle either violence or health care access but not both. A further limitation is that we did not assess the quality of the papers, selected although all were published in peer-reviewed journals.

In addition, it should also be noted that the search on which this review is based was carried out in early July 2020 and some important studies published in the intervening period between our study ending and publication may have been missed. Also, this is not a global review as we did not include papers based on data from high-income countries. It nevertheless provides a very useful body of evidence for countries which face similar developmental and health related issues.

## Conclusions and recommendations

Urban violence is a structural and systemic issue that, particularly in low-income areas in LMICs, both determines health and frames the conditions for accessing health care. It impacts on who and who does not seek health care, what type of services they seek, which services are developed and the experiences of care provided. This suggests that a multi-sectoral approach involving inter-disciplinary and cross-sector collaboration is required to minimise the negative impact of violence on youth access to health care. We would also recommend the creation of a global repository to harness knowledge and practice in the area.

There is a clear need for feasible interventions which address broader issues of violence and youth access to health care. Many interventions take a top-down approach rather than working with the communities, although violence prevention initiatives that have used community participatory approaches, for example carried out in the United States (Kia-Keating et al., 2017), provide clear evidence that community engagement is key to implementation, scale-up and sustainability in high-violence, low resource settings (Shadowen et al., 2017). This would suggest that community engagement and a multi-sectoral approach are key to ameliorate the impact of violence on youth health care access.

## Supplementary Material

Supplemental Material
