# Supplementary material for: A scoping review of how exposure to urban violence impacts youth access to sexual, reproductive and trauma health care in LMICs
Source: Glob Public Health. 2022 Aug 7;18(1):2103581. doi: 10.1080/17441692.2022.2103581 (PMC9612937; doi:10.1080/17441692.2022.2103581)
Supplement: Supplemental Material [file RGPH_A_2103581_SM4235.docx]

Appendix 1: Search terms

| Category | Search term combined with AND |
| --- | --- |
| Age | 15-24 OR youth OR teenage* OR teen OR adolescen* OR young person OR adolescence OR young adult* |
| Violence | urban violence OR rape OR gender based violence OR forced sex OR sexual violence OR police violence OR gang violence OR assault OR drug trade OR informal justice OR gangs OR ganster* OR homicide OR murder* OR discrimination* OR vigilant* OR mob justice OR criminal violence OR guns OR firearms OR gun culture OR knife crime OR theft OR robbery OR community violence OR community violence exposure OR victimi* OR alcohol AND violence OR drugs AND violence  **Exclude (NOT)**: intimate partner violence OR IPV OR suicide OR suicidal ideation OR self harm OR cyber* OR bull* |
| Outcome | sexual and reproductive care OR SRH OR injury OR emergency OR trauma AND care OR treatment OR health care access OR health care seeking OR health access OR health seeking OR adherence OR health service* OR health care provider OR health care worker* |
